# Clinical and prognostic associations of anti-Jo-1 antibody levels in patients with antisynthetase syndrome

**DOI:** 10.1186/s12931-024-02851-w

**Published:** 2024-05-29

**Authors:** Hongxia Yang, Qingning Chen, Chao Sun, Qiwen Jin, Lining Zhang, Qingyan Liu, Qinglin Peng, Guochun Wang, Xin Lu

**Affiliations:** 1 Department of Rheumatology, China-Japan Friendship Hospital, No.2 Yinghua East Road Chaoyang District, Beijing, 100029 China; 2 Department of Clinical Laboratory, Peking University First Hospital, Beijing, China; 3 Department of Clinical Nutrition, The First Affiliate Hospital of Xiamen University, Xiamen, China; 4 Peking University China-Japan Friendship School of Clinical Medicine, Beijing, China

**Keywords:** Anti-Jo-1 antibody levels, Antisynthetase syndrome, Disease activity, Prognosis

## Abstract

**Objective:**

To investigate the association of serum anti-Jo-1 antibody levels with the disease activity and prognosis in anti-Jo-1-positive patients with antisynthetase syndrome (ASS).

**Methods:**

This study included 115 anti-Jo-1-positive patients with ASS who were admitted to China-Japan Friendship Hospital between 2009 and 2019. Anti-Jo-1 antibody serum levels at initial admission and follow-up were determined by enzyme-linked immunosorbent assay (ELISA). Global and organ disease activity was assessed at baseline and follow-up according to the International Myositis Assessment and Clinical Studies guidelines.

**Results:**

Among enrolled patients, 70 (60.9%) patients initially presented with interstitial lung disease (ILD), and 46 (40%) patients presented with with muscle weakness at initial admission. At baseline, patients with ILD had lower levels of anti-Jo-1 antibodies than those without ILD (*p* = 0.012). Baseline anti-Jo-1 antibody levels were higher in patients with muscle weakness, skin involvement, and arthritis (all *p* < 0.05) compared to those without these manifestations. Baseline anti-Jo-1 antibody levels were positively correlated with skin visual analogue scale (VAS) scores (*r* = 0.25, *p* = 0.006), but not with disease activity in other organs. However, changes in anti-Jo-1 antibody levels were significantly positively correlated with the changes in PGA (β = 0.002, *p* = 0.001), muscle (β = 0.003, *p* < 0.0001), and pulmonary (β = 0.002, *p* = 0.013) VAS scores, but not with skin and joint VAS scores. Older age of onset (hazard ratio [HR] 1.069, 95% confidence interval [CI]:1.010–1.133, *p* = 0.022) and higher C-reactive protein (CRP) levels (HR 1.333, 95% CI: 1.035–1.717, *p* = 0.026) were risk factors for death.

**Conclusion:**

Anti-Jo-1 titers appear to correlate more with disease activity changes over time rather than with organ involvement at baseline, which provides better clinical guidance for assessing the disease course using anti-Jo-1 levels.

**Supplementary Information:**

The online version contains supplementary material available at 10.1186/s12931-024-02851-w.

## Introduction

Antisynthetase syndrome (ASS), a subtype of idiopathic inflammatory myopathies (IIM), manifests as one or more clinical symptoms such as interstitial lung disease (ILD), arthritis, mechanic’s hand, myositis, fever, Raynaud’s phenomenon, and the prominent occurrence of circulating antisynthetase antibodies (ASA) [1–4]. To date, more than ten ASAs targeting aminoacyl-tRNA synthetases have been reported [5–7]. Among them, the first ASA reported in 1980 was anti-histidyl-tRNA synthetase (HisRS), also known as anti-Jo-1 antibody, which was closely associated with ILD and systemic symptoms in patients with ASS [8]. Anti-Jo-1 antibody is also the most common ASA, with a positivity rate of 60–70% in ASS patients [9]. ILD, one of the most common manifestations of ASS, may be the first and sometimes the only feature of ASS [10–13]. Approximately 79–90% of anti-Jo-1-positive patients with ASS will develop ILD during the course of the disease, which can adversely affect prognosis [14, 15].

Previous studies have demonstrated that HisRS, the target antigen of anti-Jo-1 antibodies, is overexpressed in lung and muscle tissue and that pro-inflammatory His-tRNA synthetase-specific CD4^+^ T cells are present in the lungs and circulation of these patients. Therefore, HisRS may be involved in the pathogenesis of lung and muscle involvement in ASS [16–18]. Furthermore, the levels of anti-Jo-1 antibody have been shown to be associated with the disease activity in ASS patients in cross-sectional and longitudinal analyses [19, 20]. Paradoxically, some studies have suggested that anti-Jo-1 antibody levels are associated with the activity of specific organs (i.e., lungs, muscles, and joints), while others have found no correlation between anti-Jo-1 antibody levels and disease activity [21]. However, the relationship between anti-Jo-1 antibody levels and disease and organ activity has not been fully elucidated, so the exact value of antibody detection levels in disease surveillance remains to be determined.

Therefore, this study aimed to investigate the association between serum levels of anti-Jo-1 antibodies and disease activity and to evaluate the impact of anti-Jo-1 antibody levels on the prognosis of ASS patients.

## Methods

### Patient enrollment

All patients diagnosed with ASS according to the 2010 Conner’s criteria and also positive for anti-Jo-1 antibodies who were admitted to the Department of Rheumatology and Immunology of China-Japan Friendship Hospital from 2009 to 2019 were included in this study [3].

All patients completed informed consent forms, and the study was approved by the Research Review Committee and the Ethical Review Committee of the China-Japan Friendship Hospital (approval number 2019-SDZL-3). This study was conducted in accordance with the tenets of the Declaration of Helsinki.

### Data collection and clinical assessment

Clinical information, including patient demographic data, laboratory test results, and imaging findings, was retrospectively collected at baseline and during follow-up. The diagnosis of ILD was determined based on high-resolution chest computed tomography and abnormal pulmonary function tests [22]. Skin involvement included Mechanic’s hand, Gottron sign, heliotrope sign, and Raynaud’s phenomena. Patients disease activity was assessed at baseline and each follow-up visit. The global and organ disease activities were assessed using Core Set Measures conducted by the International Myositis Assessment and Clinical Studies Group, including the physician’s global assessment (PGA) on 10-cm visual analogue scale (VAS) and pulmonary, muscle, skin, and joint VAS, in all enrolled patients [23–25]. The survival status and cause of death were followed up by telephone and medical records, and the follow-up period was defined as the time from diagnosis to the date of death or last visit.

### Detection of anti-Jo-1 antibodies

The patients’ sera from the first and follow-up visits were collected and stored at -80 °C. A human Jo-1 quantitative enzyme-linked immunosorbent assay (ELISA) kit (Inova Diagnostics, California, USA) was used to detect the levels of anti-Jo-1 antibodies according to the manufacturer’s instructions, and levels > 15 IU/L were considered positive. Line blot (Euroimmun, Lübeck, Germany) for myositis-specific and myositis-associated antibodies were used to confirm positive anti-Jo-1 antibodies.

### Statistical analysis

All statistical analyses were performed using GraphPad Prism 7.0 (GraphPad, San Diego, CA, USA) and SPSS version 26 (IBM Corp., Armonk, NY, USA). Numerical variables are expressed as medians and interquartile ranges, and categorical variables are expressed as numbers and proportions. Differences in non-normally distributed groups of numerical variables were tested using the Mann–Whitney U test, and differences in categorical variables were analyzed using the chi-square or Fisher’s exact test, as appropriate. Spearman’s rank correlation was used to determine cross-sectional correlations. Generalized estimating equations (GEE) were used to analyze longitudinal data. Univariate and multivariate COX analyses were used to analyze risk factors affecting mortality. The area under the receiver operating characteristics (ROC) curve was used to determine the power of the univariate and multivariate Cox models to assess their reliability in predicting risk factors for mortality, and *p* < 0.05 was considered statistically significant.

## Results

### Clinical characteristics of anti-Jo-1 autoantibody-positive patients

The study included 115 patients who were positive for anti-Jo-1 on first admission. Anti-Jo-1 antibody positivity was determined by both ELISA and line blot. The enrolled patients were 85 women (73.9%) and 30 men (26.1%), with an age of onset of [52 (41,61; IQR)] years and a disease duration of [9(3,36); IQR] months. At baseline, 70 patients (60.8%) initially presented with ILD, 46 patients (40.0%) with muscle weakness, 46 (40%) with skin involvement, and 58 (50.4%) with arthritis. Based on lung and muscle involvement at baseline, 115 patients included 52 patients (45.2%) with isolated ILD, 28 (24.3%) patients with isolated myositis, 18 (15.7%) patients with both and 17 (14.8%) patients with neither. Anti-Jo-1 antibody levels were as 135 (98,185) U/ml at baseline for all patients. During the course of disease, 112 (97.4%) patients developed ILD, 52 (45.2%) with muscle weakness, 75 (65.2%) with skin involvement, and 64 (55.7%) with arthritis (Table 1).

**Table 1:** Characteristics of anti-Jo-1-positive patients

**Variables**	**Overall(** ***n*** **=115)**
Female	85(73.9%)
Age at onset, years	52(41,61)
Duration, months	9(3,36)
Treatment naive	43(37.4%)
Manifestation at baseline	
ILD	70(60.8%)
Muscle weakness	46(40.0%)
Skin involvement	46(40.0%)
Arthritis	58(50.4%)
Isolated ILD	52(45.2%)
Isolated myositis	28(24.3%)
Both ILD and myositis	18(15.7%)
Neither ILD nor myositis	17(14.8%)
Laboratory findings at baseline	
ANA (>1:160)	28(24.3%)
ALT (IU/L)	30(17,59)
AST(IU/L)	25(17,50)
LDH (IU/L)	288(219,394)
CK (U/mL)	140(49,671)
IgG(mg/dl)^b^	1230(1020,1640)
IgA(mg/dl)^b^	234(173,304)
IgM(mg/dl)^b^	123(89,192)
ESR (mm/h)	13(6,24)
CRP (mg/dl)	0.44(0.21,1.55)
Ferritin(0-306.7ng/ml)	111(61,237)
Anti-Jo-1 levels(U/ml)	135(98,185)
Manifestation during follow-up	
ILD	112(97.4%)
Muscle weakness	52(45.2%)
Myalgia	40(34.8%)
Dysphagia	7(6.1%)
Dyspnea	68(59.1%)
Skin involvement	75(65.2%)
Mechanic’s hands	50(43.5%)
Gottron sign	29(25.2%)
Heliotrope sign	23(20%)
Raynaud’s phenomena	6(5.2%)
Arthritis/arthralgia	64(55.7%)
Fever	34(29.6%)
Malignancy	7(6.1%)
With other CTD	16(13.9%)
Myositis associated antibodies^a^	
Anti-Ro-52	60(52.2%)
Anti-Ku	2(1.7%)
Anti-PM-Scl75/100	1(0.9%)
Anti-AMA-M2	3/113(2.6%)
Pulmonary function tests	
%FVC^c^	76.73±19.54
%FEV1^c^	73.55±18.21
%DLco^d^	58.57±19.51

*Abbreviations:* *ILD* interstitial lung disease, *ANA* antinuclear antibodies, *ALT* alanine aminotransferase, *AST* aspartate aminotransferase, *LDH* lactic dehydrogenase, *CK* creatine kinase, *ESR* erythrocyte sedimentation rate, *CRP* C reactive protein, *CTD* connective tissue disease, *%FVC* percent predicted forced vital capacity, *%FEV1* percent predicted forced expiratory volume in one second, *%DLco* percent predicted carbon monoxide diffusion capacity

^a^coexistence with anti-Jo-1 antibodies and MAAs

^b﻿^Data available for 111 patients

^c﻿^Data available for 71 patients

^d﻿^Data available for 69 patients

### Association of anti-Jo-1 antibody levels with different organ involvement

**Fig. 1:**
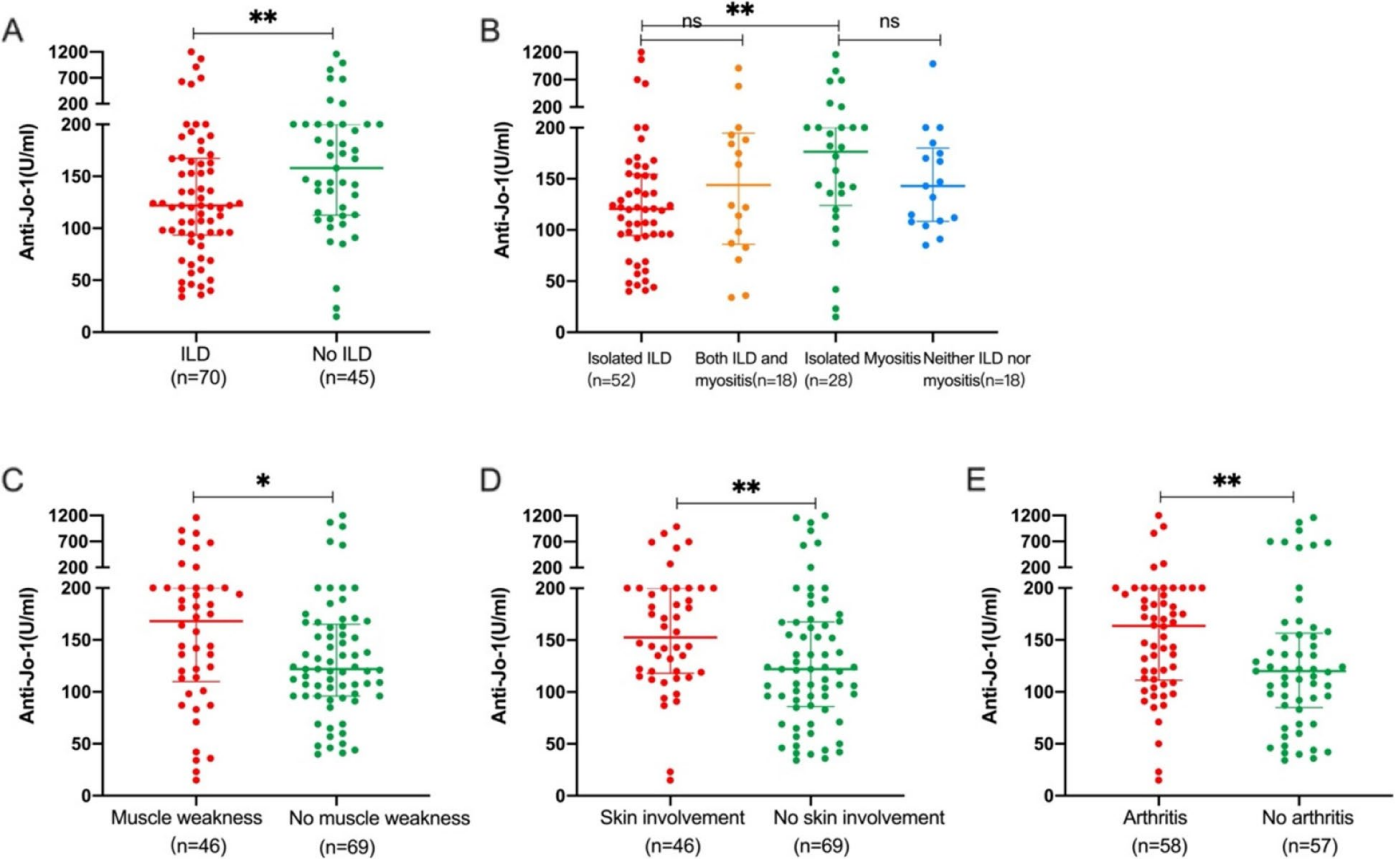
The serum levels of anti-Jo-1 in patients with anti-synthetase syndrome. **A** Anti-Jo-1 levels in patients with and without ILD at baseline [122 (94, 167) vs. 158 (113, 200) U/ml, *p* = 0.012]; **B** Anti-Jo-1 levels in patients with isolated ILD [121(95,155)], both ILD and myositis [144(86,195)], isolated myositis [177(124,200)], and neither ILD nor myositis [143(109,180)]; **C** Anti-Jo-1 levels in patients with and without muscle weakness at baseline[168(110, 200) vs. 122 (96, 165) U/ml, *p* = 0.018]; **D** Anti-Jo-1 levels in patients with and without skin involvement on the first admission [153(118, 200) vs. 122 (86, 168) U/ml, *p* = 0.009]; **E** Anti-Jo-1 levels in patients with and without arthritis at baseline [164 (111, 200) vs. 120 (85, 157) U/ml, *p* = 0.006]. *p < 0.05; **p < 0.01

When comparing the serum levels of anti-Jo-1 antibody levels at baseline between patients with different symptoms on admission, patients with ILD at baseline had lower levels of anti-Jo-1 antibodies than those without ILD at baseline [122 (94, 167) vs. 158 (113, 200) U/ml, *p* = 0.012] (Fig. 1A). When comparing anti-Jo-1 levels in the four groups of patients with both ILD and myositis, isolated ILD, isolated myositis, and neither, a statistically significant difference was found only in the isolated myositis and isolated ILD groups [177(124,200) vs. 121(95,155), *p* < 0.001] (Fig. 1B). Patients with isolated myositis had higher baseline anti-Jo-1 levels than those with isolated ILD [177 (124,200) vs. 121(95,155) U/ml, *p* = 0.004] (Fig. 1B). Baseline anti-Jo-1 antibody levels were higher in patients with muscle weakness [168(110, 200) vs. 122 (96, 165) U/ml, *p* = 0.018] (Fig. 1C), skin involvement [153(118, 200) vs. 122 (86, 168) U/ml, *p* = 0.009] (Fig. 1D), and arthritis [164 (111, 200) vs. 120 (85, 157) U/ml, *p* = 0.006] (Fig. 1E) compared to those without these manifestations. We also compared anti-Jo-1 levels in ASS with and without anti-Ro52, as well as in treated and treatment naïve patients. There was no difference in anti-Jo-1 antibody levels in patients with and without anti-Ro-52 [139(110,192) vs. 120(82,181), *p* = 0.112], and treated and treatment naive patients [129(96,182) vs. 136(105,199), *p* = 0.527] (Supplementary Tables 1 and 2).

Anti-Jo-1 levels in patients with new development of organ involvement were also analyzed. Anti-Jo-1 levels were higher in patients with new development of skin involvement than those without new development of skin involvement [136 (101, 191) vs. 107 (66, 155) U/ml, *p* = 0.032], but no statistical difference was found in new development of other organ involvement (Supplementary Figure 1).

### Correlation between anti-Jo-1 autoantibody levels and disease activity

**Fig. 2:**
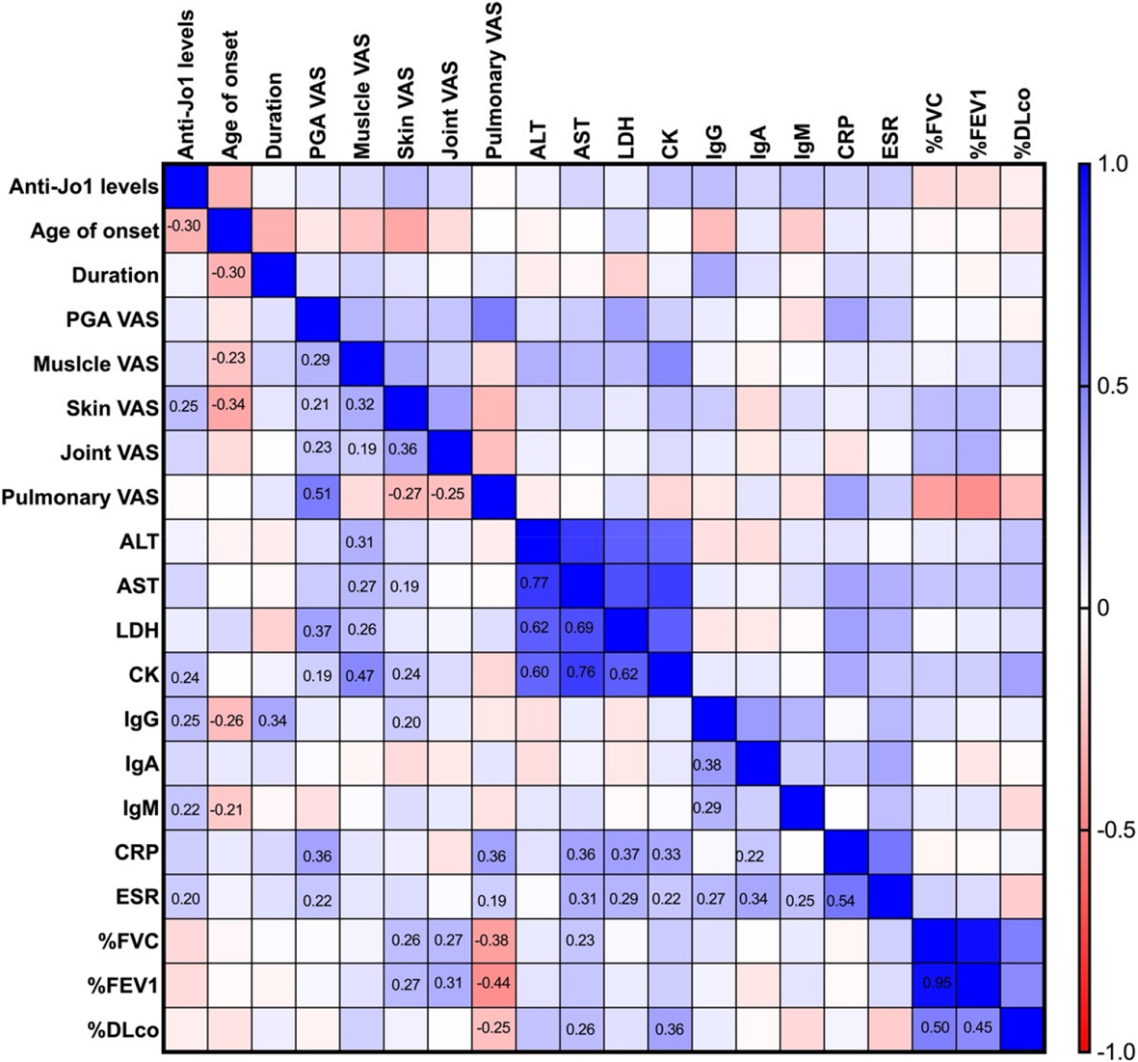
Correlation matrix of baseline anti-Jo-1 levels, laboratory test results, and disease activity. Spearman’s correlation analysis was used to analyze the correlation between the variables. The numbers in the boxes represent the correlation coefficient between the two variables (red negative, blue positive) when *p* < 0.05. PGA, Physician’s Global Assessment; VAS, Visual Analog Scale; ALT, alanine aminotransferase; AST, aspartate aminotransferase; LDH, lactic dehydrogenase; CK, creatine kinase; CRP, C reactive protein; ESR, erythrocyte sedimentation rate; %FVC, percent predicted forced vital capacity; %FEV1, percent predicted forced expiratory volume in one second; %DLco, percent predicted carbon monoxide diffusion capacity

In the overall cohort, the anti-Jo-1 levels were negatively correlated with the age of onset (*r *=-0.30, *p* = 0.001). Baseline anti-Jo-1 antibody levels of patients were positively correlated with skin VAS score (*r* =0.25, *p* = 0.006), but were not correlated with PGA, muscle, pulmonary, or joint VAS scores. In addition, the baseline anti-Jo-1 antibody levels were positively associated with levels of CK (*r* = 0.24, *p* = 0.011), IgG (*r* = 0.25, *p* = 0.008), IgM (*r* = 0.22, *p* = 0.019), and erythrocyte sedimentation rate (ESR; *r* = 0.20, *p* = 0.035). No correlation was found between anti-Jo-1 antibody levels and lung function (Fig. 2).

The correlation between anti-Jo-1 levels and disease activity in different subgroups were shown in Supplementary Table 3 (with and without ILD and baseline), Supplementary Table 4 (with and without anti-Ro-52) and Supplementary Table 5 (treated and treatment naive), and no significant correlation was found.

### Longitudinal correlation between anti-Jo-1 autoantibody levels and disease activity

**Fig. 3:**
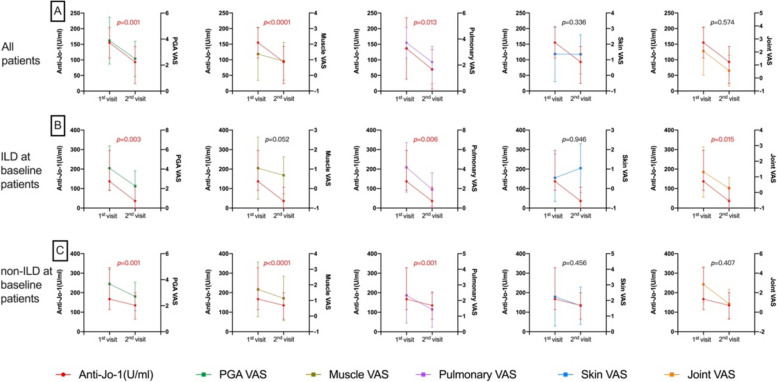
Longitudinal relationship between anti-Jo-1 levels and the disease activity in all anti-Jo-1 patients (**A**), anti-Jo-1 patients with ILD at baseline (**B**), and anti-Jo-1 patients with non-ILD at baseline (**C**). LD interstitial lung disease, PGA Physician’s Global Assessment, VAS Visual Analog Scale

Ninety-eight of the 115 patients were followed up to survival status, with 1–9 visits. The follow-up disease activity and anti-Jo-1 antibody levels were examined in 43 patients with two or more follow-up visits. The longitudinal correlation between anti-Jo-1 antibody levels and disease activity was further estimated using GEE models. Overall, changes in anti-Jo-1 antibody levels were significantly positively correlated with the changes in PGA (β = 0.002, *p* = 0.001), muscle (β = 0.003, *p* < 0.0001), and pulmonary (β = 0.002, *p* = 0.013) VAS scores, but not with skin and joint VAS scores. Meanwhile, changes in anti-Jo-1 antibody levels were also positively correlated with changes in the levels of AST, CK, IgG, IgM (all *p* < 0.05) and negatively correlated with changes in the percent predicted forced vital capacity (%FVC; β = -0.025, *p* < 0.0001), percent predicted forced expiratory volume in one second (%FEV1; β = -0.021, *p* < 0.0001) (Fig. 3 and Supplemental Table 6).

In patients with ILD at baseline, changes in anti-Jo-1 antibody levels were correlated with changes in PGA, pulmonary, and joint VAS scores; IgG, IgA, IgM, and CRP levels; %FVC; %FEV1; and percent predicted carbon monoxide diffusion capacity (%DLco) (all *p* < 0.05). Variations in anti-Jo-1 antibody levels were significantly correlated with changes in PGA, muscle, and pulmonary VAS scores; ALT, CK, IgG, and IgA levels; %FVC, and %FEV1 in patients without ILD at baseline (all *p* < 0.05) (Table 2).

**Table 2:** Univariate and multivariate Cox proportional hazard models of death in anti-Jo-1-positive patients

Findings, number (%)	Univariate cox analysis			Multivariate cox analysis		
	*p* value	Hazard ratio	95%CI	*p* value	Hazard ratio	95%CI
Female	0.426	1.864	0.402–8.650			
Age at onset, years	0.006	1.074	1.021–1.129	0.022	1.069	1.010–1.133
Duration, months	0.518	0.992	0.9701.015			
ILD at baseline	0.130	3.271	0.705–15.174			
Muscle weakness at baseline	0.285	1.913	0.583–6.279			
Skin involvement at baseline	0.517	0.644	0.170–2.438			
Arthritis at baseline	0.031	0.184	0.039–0.855	0.063	4.925	0.917–26.46
ANA (> 1:160)	0.134	1.005	0.999–1.011			
Anti-Ro52	0.718	0.789	0.218–2.850			
ALT (IU/L)	0.505	1.004	0.993–1.014			
AST(IU/L)	0.243	1.004	0.997–1.012			
LDH (IU/L)	0.380	1.001	0.999–1.003			
CK (U/mL)	0.759	1	0.999-1			
IgG(mg/dl)	0.427	0.999	0.989–1.001			
IgA(mg/dl)	0.018	1.006	1.001–1.010	0.140	1.004	0.998–1.009
IgM(mg/dl)	0.185	0.993	0.982–1.003			
CRP (mg/dl)	0.025	1.338	1.037–1.726	0.026	1.333	1.035–1.717
ESR (mm/h)	0.051	1.026	1.000-1.054			
Ferritin(ng/ml)	0.309	1.002	0.998–1.005			
Anti-Jo-1 levels	0.313	0.997	0.992–1.003			

*Abbreviations:* *ILD* interstitial lung disease, *ANA* antinuclear antibodies, *ALT* alanine aminotransferase, *AST* aspartate aminotransferase, *LDH* lactic dehydrogenase, *CK* creatine kinase, *CRP* C reactive protein, *ESR* erythrocyte sedimentation rate

**Fig. 4:**
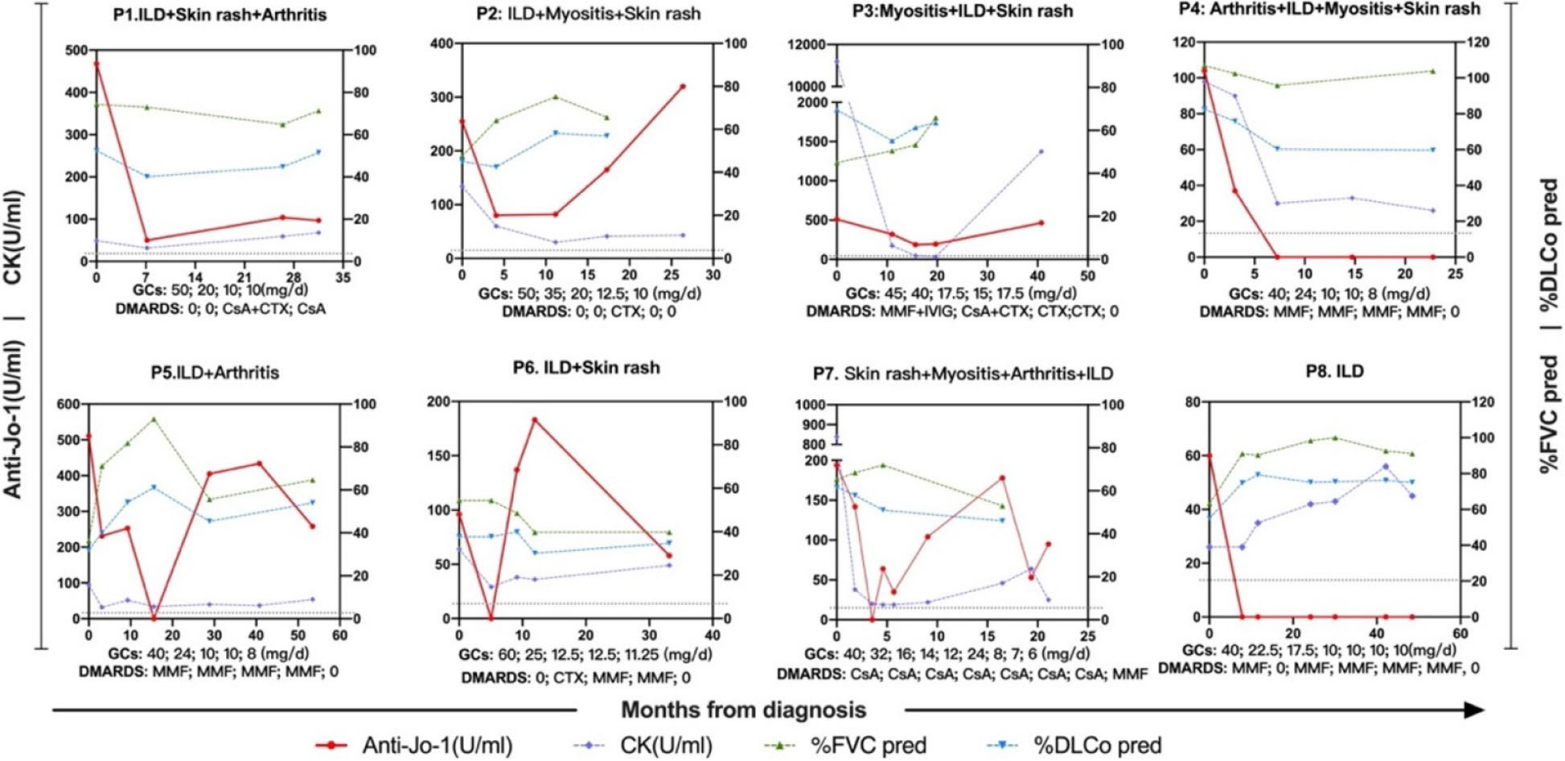
Longitudinal changes of anti-Jo-1 levels in and serum CK levels, %FVC pred, and %DLco pred over time in 8 anti-Jo-1-positive patients with more than 3 times visits. The black dashed line indicates the cut-off line for the normal level of anti-Jo-1 antibodies(15U/ml). Abbreviation: ILD, interstitial lung diseases; CK, creatine kinase; %FVC, percent predicted forced vital capacity; %DLco, percent predicted carbon monoxide diffusion capacity; GCs, corticosteroids steroids; DMARDS, disease-modifying anti-rheumatic drugs; CsA, ciclosporin A; CTX, cyclophosphamide; MMF, mycophenolate mofetil; IVIG, intravenous. immunoglobulin; tac, tacrolimus

Among 43 patients with two or more follow-up visits, 8 who were anti-Jo-1-positive had more than 3 visits. The variability of anti-Jo-1 antibody and serum CK levels, %FVC, and %DLco over time in these patients showed comparable trends over time in the majority of patients (Fig. 4).

### Association between anti-Jo-1 antibody levels and prognosis in patients with ASS

A total of 98 anti-Jo-1 positive patients were followed up for 1 month to 118.87 months, and the median follow-up time was 41.89 months. Eleven deaths were observed, including nine from respiratory failure, one from endometrial cancer, and one from malignancies.

Risk factors for mortality in anti-Jo-1 positive patients were analyzed. Univariate analyses showed that older age of onset (HR 1.074, 95% CI: 1.021–1.129, *p* = 0.006), arthritis at baseline(HR 0.184, 95% CI: 0.039–0.855, *p *= 0.031), and higher levels of IgA (HR 1.006, 95% CI: 1.001–1.010, *p* = 0.018) and CRP (HR 1.338, 95% CI: 1.037–1.726, *p* = 0.025) were risk factors for death in anti-Jo-1-positive patients. Further multivariate Cox regression analysis showed that a higher age of onset (HR 1.069, 95% CI: 1.010–1.133, *p* = 0.022), and higher CRP levels (HR 1.333, 95% CI: 1.035–1.717, *p* = 0.026) were risk factors for death in anti-Jo-1-positive patients. Patients who died had an older age of onset [61 (48, 79) vs. 50 (39, 61) years old, *p* = 0.009] and higher CRP levels [1.55 (0.41, 2.90) vs. 0.43 (0.21, 1.30) mg/dl, *p* = 0.039] than those who survived. Baseline anti-Jo-1 levels were not risk factors for death in anti-Jo-1-positive patients (*p* = 0.997) (Table 2). The multivariate cox model, which included age of onset and CRP, showed the best efficacy for predicting death, with the largest area under the ROC curve [0.770(0.625–0.915)] (Supplementary Table 6).

## Discussion

This study demonstrated the clinical associations and the prognostic significance of anti-Jo-1 antibodies in patients with ASS. We found that baseline anti-Jo-1 levels were lower in patients with ILD at baseline, but higher in patients with extrapulmonary involvement at baseline. Anti-Jo-1 antibody levels were weakly correlated with disease activity in the cross-sectional analysis and were not related with the final outcome. In addition, a smaller longitudinal subset follow-up analysis showed that anti-Jo-1 levels reflected the disease activity to some extent.

Myositis, ILD, and arthritis are common manifestations of ASS, and the spectrum of symptoms in different patients can be distinct. Previous studies have shown that 51-55.2% of Jo-1-positive patients have ILD at onset, and 82-84% of patients will eventually develop ILD during their clinical course [10, 11, 26]. In this study, we found that anti-Jo-1 levels were higher in patients with muscle weakness, skin involvement, and arthritis, but lower in patients with ILD. A previous study found that patients with dermatomyositis had lower levels of anti-Jo-1 antibody than those with polymyositis (127 vs. 178 IU/l). Although differences in anti-Jo-1 antibody levels in different subgroups were not analyzed, the results seem to suggest that anti-Jo-1-positive patients with different phenotypes have different anti-Jo-1 antibody levels [21].

We found no statistically difference in the proportion of receiving treatment in patients with and without ILD (60% vs. 66.7%, *p* = 0.471). Thus, ILD patients with lower baseline anti-Jo-1 levels may not be related to whether they receive treatment, which may be related to the differences in antigen levels and antibody types in different tissues. The antigen recognized by the anti-Jo-1 antibody is HisRS, which consists of 509 amino acids and can be divided into three domains: WHEP, internal catalytic domain (CD), and anticodon binding domain (ABD) [27]. Additionally, splice variants of HisRS that skip CD and link the noncatalytic domain, HisRS∆CD, also exist in serum and tissues, with particularly enriched expression in human lung tissue. Recent studies discovered that the serum from anti-Jo-1-positive patients had different affinities for different antigen fragments of HisRS [27]. The anti-Jo-1 antibody ELISA kit we used in this study was a commercial kit coated with a recombinant HisRS full-length (HisRS-FL), which could detect the levels of anti-Jo-1 antibody that react with HisRS-FL. Research by Zhou et al. found that the tissue distribution of HisRS-FL and HisRS^WHEP^ was different, with HisRS^WHEP^ having higher expression in the lung than HisRS-FL, while HisRS-FL was predominantly expressed in muscle, which had very low levels of HisRS^WHEP^ [28]. This may explain the differences in baseline anti-Jo-1 antibody levels in subgroups involving different organs. In patients with muscle damage, exposure to HisRS-FL in muscle tissue induced the production of anti-Jo-1 antibodies, resulting in higher levels of anti-Jo-1 antibodies in patients with muscle weakness. However, anti-Jo-1 antibodies mainly against HisRS^WHEP^ may not be detected by this kit in patients with ILD, and the higher percentage of muscle weakness in the non-ILD subgroup (60% vs. 25.7%, *P* < 0.0001) may also contribute to the higher levels of anti-Jo-1 antibodies in patients without ILD.

In this study, anti-Jo-1 antibody levels were only weakly correlated with skin VAS scores in the cross-sectional analysis, but no correlation with anti-Jo-1 antibody levels or other organ VAS scores was found. Stone et al. found a modest correlation between the anti-Jo-1 antibody and CK levels and muscle and joint disease activity in a cross-sectional assessment of a cohort of 94 anti-Jo-1-positive patients [13, 21], which is not entirely consistent with the findings of this study. There was also a correlation between anti-Jo-1 antibody levels and some laboratory parameters (CK, IgG, IgM, and ESR). Kryštůfková et al. also found a correlation between the anti-Jo-1 antibody level and levels of CK, AST, and CRP [21]. These findings indicate that cross-sectional anti-Jo-1 antibody levels may not have good correlation with disease activity.

In the follow-up study, changes in anti-Jo-1 levels were correlated with changes in most parameters (PGA, pulmonary, and skin VAS scores; AST, CK, IgG, and IgM levels; and %FVC), and previous studies have shown similar findings. We also found that the spectrum of variables associated with changes in anti-Jo-1 antibody levels differed in different subgroups. Changes in anti-Jo-1 antibody levels were not associated with changes in muscle enzymes but were associated with changes in parameters related to lung disease severity (pulmonary VAS score, %FVC, and %DLco) in patients with ILD at baseline. In addition, changes in anti-Jo-1 antibody levels were correlated with changes in indicators of the severity of muscle damage (muscle VAS score, ALT level, and CK level) in patients without ILD at baseline. This suggests that longitudinal anti-Jo-1 antibody levels may better reflect disease activity in the damaged target organs. The anti-Jo-1 antibody recruits T lymphocytes and damages target organs (lung and muscle), which is one of the reasons for the pathogenicity of Jo-1 antibody-positive ASS [16, 29]. This may explain the correlation between anti-Jo-1 levels and disease activity in the target organs.

Subsequently, we analyzed the risk factors affecting the outcome of anti-Jo-1-positive patients and found that baseline anti-Jo-1 antibody levels were not a risk factor for death. In cross-sectional analyses, we were also unable to find an association between the level of anti-Jo-1 antibodies and the activity of the disease. This suggests that pre-post changes in anti-Jo-1 antibody levels may be more indicative of disease activity than their absolute levels. Therefore, focusing on longitudinal changes in anti-Jo-1 antibody levels may be more important in clinical practice. In addition, an older age of onset and high CRP levels were risk factors for death. In contrast, the main cause of death was ILD-related respiratory failure (9/11, 81.8%). Patients who died had a higher pulmonary VAS score at baseline than those who survived (2.53 ± 0.76 vs. 2.01 ± 0.21, *P* = 0.043), and pulmonary VAS scores at baseline were positively associated with the age of onset and CRP levels, which may explain why patients with an older age of onset and higher CRP levels had a lower survival rate. Consistent with our results, Wei Jiang et al. also reported that ASS patients over 60 years of age had a lower survival rate [30].

This study had some limitations. First, we determined anti-Jo-1 antibody positivity by ELISA and immunoblotting, but we did not use immunoprecipitation for confirmation. Although it is possible that some individual patients may have had false positives, especially those with low titers, this may not have affected the main conclusions of this study. Second, some patients were treated at enrolment, and the treatment may affect antibody levels and disease status. Finally, our longitudinal association analysis was performed in only a small subset of patients due to data availability, which may have introduced bias.

In conclusion, this study revealed that levels of anti-Jo-1 antibodies were weakly associated with disease activity at the cross-sectional level and with final outcome. However, longitudinal changes of anti-Jo-1 antibody levels reflect the disease activity and may be more strongly associated with disease activity in the major affected organs, which may provide clinicians with more effective guidance in assessing disease progression based on anti-Jo-1 antibody levels.

### Supplementary Information

Supplementary Material 1.

Supplementary Material 2.

## Data Availability

The data that support the findings of this study are not openly available due to reasons of sensitivity and are available from the corresponding author upon reasonable request.
