# Supplementary material for: Clinical and prognostic associations of anti-Jo-1 antibody levels in patients with antisynthetase syndrome
Source: Respir Res. 2024 May 29;25:222. doi: 10.1186/s12931-024-02851-w (PMC11137886; doi:10.1186/s12931-024-02851-w)
Supplement: Supplementary file 1 — Supplementary Material 1. [file 12931_2024_2851_MOESM1_ESM.docx]

**Supplement material**

**Supplementary Table 1** General features of anti-Jo-1-positive patients with and without anti-Ro52 autoantibodies

| **Variables** | **Overall(n=115)** | **Ro-52+(n=60)** | **Ro-52-(n=55)** | ***p* value** |
| --- | --- | --- | --- | --- |
| Female | 85(73.9%) | 45(75%) | 40(72.7%) | 0.782 |
| Age at onset, years | 52(41,61) | 51(38,62) | 52(43,61) | 0.689 |
| Duration, months | 9(3,36) | 11(4,39) | 9(2,24) | 0.164 |
| Treatment naive | 43(36.5%) | 23(41.8%) | 20(33.3%) | 0.348 |
| Manifestation at baseline |  |  |  |  |
| ILD | 70(60.8%) | 39(65%) | 31(56.4%) | 0.343 |
| Muscle weakness | 46(40.0%) | 25(41.7%) | 21(38.3%) | 0.703 |
| Skin involvement | 46(40.0%) | 28(46.7%) | 18(32.7%) | 0.127 |
| Arthritis/arthralgia | 58(50.4%) | 29(48.3%) | 29(52.7%) | 0.638 |
| Laboratory findings at baseline |  |  |  |  |
| ANA (>1:160) | 28(24.3%) | 19(31.7%) | 9(16.4%) | 0.056 |
| ALT (0-40 IU/L) | 30(17,59) | 30(16,46) | 30(22,72) | 0.267 |
| AST(0-40IU/L) | 25(17,50) | 26(19,38) | 22(17,58) | 0.923 |
| LDH (100-250IU/L) | 288(219,394) | 290(232,415) | 263(207,376) | 0.234 |
| CK (26-200U/mL) | 140(49,671) | 160(56,579) | 108(41,1186) | 0.995 |
| Anti-Jo-1 levels(U/ml) | 135(98,185) | 139(110,192) | 120(85,181) | 0.122 |
| Manifestation during follow-up |  |  |  |  |
| ILD | 109(94.8%) | 56(93.3%) | 53(96.4%) | 0.681 |
| Muscle weakness | 52(45.2%) | 27(45%) | 25(45.5%) | 0.961 |
| Myalgia | 40(34.8%) | 21(35%) | 19(34.5%) | 0.959 |
| Dysphagia | 7(6.1%) | 2(3.3%) | 5(9.1%) | 0.257 |
| Dyspnea | 68(59.1%) | 34(56.7%) | 34(61.8%) | 0.704 |
| Skin involvement | 74(64.3%) | 41(68.3%) | 33(60%) | 0.351 |
| Arthritis/arthralgia | 64(55.7%) | 33(55%) | 31(56.4%) | 0.833 |
| Fever | 34((29.6%) | 18(30%) | 16(29.1%) | 0.915 |
| Malignancy | 7(6.1%) | 3(5%) | 4(7.3%) | 0.708 |
| With other CTD | 16(13.9%) | 9(15%) | 7(13%) | 0.755 |
| Myositis associated antibodies |  |  |  |  |
| Anti-Ku | 2(1.7%) | 2(3.3%) | 0 | 0.497 |
| Anti-PM-Scl75/100 | 1(0.9%) | 1(1.7%) | 0 | 1 |
| Anti-AMA-M2 | 3/113(2.6%) | 1/60(1.7%) | 2/53(3.8%) | 0.599 |
| Pulmonary function tests |  |  |  |  |
| %FVC † | 76.73±19.54 | 73.91±18.26 | 79.81±20.68 | 0.229 |
| %FEV1† | 73.55±18.21 | 71.66±17.18 | 75.62±19.32 | 0.303 |
| %DLco ‡ | 58.57±19.51 | 59.37±15.73 | 57.75±22.98 | 0.670 |
| Disease activity |  |  |  |  |
| PGA VAS | 3.42±1.67 | 3.40±1.49 | 3.44±1.86 | 0.731 |
| Muscle VAS | 0.90±1.24 | 0.85±1.22 | 0.96±1.26 | 0.590 |
| Pulmonary VAS | 2.49±2.05 | 2.47±1.89 | 2.51±2.24 | 0.585 |
| Skin VAS | 1.73±1.60 | 1.97±1.59 | 1.47±1.59 | 0.126 |
| Joint VAS | 1.21±1.54 | 1.10±1.49 | 1.33±1.60 | 0.418 |

Abbreviation: ILD, interstitial lung disease; ANA, antinuclear antibodies; ALT, alanine aminotransferase; AST, aspartate aminotransferase; LDH, lactic dehydrogenase; CK, creatine kinase; ESR, erythrocyte sedimentation rate; CRP, C reactive protein; CTD, connective tissue disease; %FVC, percent predicted forced vital capacity; %FEV1, percent predicted forced expiratory volume in one second; %DLco, percent predicted carbon monoxide diffusion capacity; VAS, Visual Analog Scale.

† Data available for 71 patients.

‡ Data available for 69 patients.

**Supplementary Table 2** Comparison of general features of treated versus treatment-naïve anti-Jo-1-positive patients

| **Variables** | **Overall(n=115)** | **Treated(n=72)** | **Treatment naive(n=43)** | ***p* value** |
| --- | --- | --- | --- | --- |
| Female | 85(73.9%) | 52(72.2%) | 33(76.7%) | 0.593 |
| Age at onset, years | 52(41,61) | 49(40,80) | 55(42,64) | 0.104 |
| Duration, months | 9(3,36) | 14(6,48) | 3(1,10) | <0.0001 |
| Manifestation at baseline |  |  |  |  |
| ILD | 70(60.8%) | 42(58.3%) | 28(65.1%) | 0.471 |
| Muscle weakness | 46(40.0%) | 30(41.7%) | 16(37.2%) | 0.637 |
| Skin involvement | 46(40.0%) | 30(41.7%) | 16(37.2%) | 0.637 |
| Arthritis/arthralgia | 58(50.4%) | 33(45.8%) | 25(58.1%) | 0.202 |
| Laboratory findings at baseline |  |  |  |  |
| ANA (>1:160) | 28(24.3%) | 19(26.4%) | 9(20.9%) | 0.509 |
| ALT (IU/L) | 30(17,59) | 49(17,46) | 39(16,71) | 0.264 |
| AST(IU/L) | 25(17,50) | 21(17,42) | 30(18,65) | 0.025 |
| LDH (IU/L) | 288(219,394) | 225(191,340) | 309(242,461) | 0.015 |
| CK (U/mL) | 140(49,671) | 131(39,441) | 220(65,1376) | 0.047 |
| Anti-Jo-1 levels(U/ml) | 135(98,185) | 136(105,199) | 129(96,182) | 0.527 |
| Manifestation during follow-up |  |  |  |  |
| ILD | 109(94.8%) | 70(97.2%) | 39(90.7%) | 0.194 |
| Muscle weakness | 52(45.2%) | 33(45.8%) | 19(44.2%) | 0.864 |
| Myalgia | 40(34.8%) | 22(30.65) | 18(41.9%) | 0.218 |
| Dysphagia | 7(6.1%) | 4(5.6%) | 3(7%) | 1 |
| Dyspnea | 68(59.1%) | 44(61.1%) | 24(55.8%) | 0.576 |
| Skin involvement | 74(64.3%) | 43(59.7%) | 31(72.1%) | 0.180 |
| Arthritis/arthralgia | 64(55.7%) | 37(51.4%) | 27(62.8%) | 0.234 |
| Fever | 34((29.6%) | 20(27.8%) | 14(32.6%) | 0.587 |
| Malignancy | 7(6.1%) | 4(5.5%) | 3(7%) | 0.758 |
| With other CTD | 16(13.9%) | 1318.1%) | 3(7.1%) | 0.106 |
| Myositis associated antibodies |  |  |  |  |
| Anti-Ro-52 | 60(52.2%) | 40(55.6%) | 20(46.5%) | 0.391 |
| Anti-Ku | 2(1.7%) | 0 | 2(4.7%) | 0.138 |
| Anti-PM-Scl75/100 | 1(0.9%) | 1(1.4%) | 0 | 0.438 |
| Anti-AMA-M2 | 3/113(2.6%) | 1(1.4%) | 2(4.7%) | 0.301 |
| Pulmonary function tests |  |  |  |  |
| %FVC † | 76.73±19.54 | 75.77±16.18 | 78.29±24.32 | 0.463 |
| %FEV1† | 73.55±18.21 | 72.04±14.16 | 76.02±23.48 | 0.365 |
| %DLco ‡ | 58.57±19.51 | 57.30±20.00 | 60.82±18.79 | 0.803 |
| Disease activity |  |  |  |  |
| PGA VAS | 3.42±1.67 | 3.11±1.30 | 3.93±2.08 | 0.055 |
| Muscle VAS | 0.90±1.24 | 0.83±1.18 | 1.02±1.34 | 0.481 |
| Pulmonary VAS | 2.49±2.05 | 2.19±1.66 | 2.98±2.53 | 0.434 |
| Skin VAS | 1.73±1.60 | 1.61±1.68 | 1.93±1.45 | 0.242 |
| Joint VAS | 1.21±1.54 | 0.82±1.37 | 1.86±1.61 | <0.0001 |

Abbreviation: ILD, interstitial lung disease; ANA, antinuclear antibodies; ALT, alanine aminotransferase; AST, aspartate aminotransferase; LDH, lactic dehydrogenase; CK, creatine kinase; ESR, erythrocyte sedimentation rate; CRP, C reactive protein; CTD, connective tissue disease; %FVC, percent predicted forced vital capacity; %FEV1, percent predicted forced expiratory volume in one second; %DLco, percent predicted carbon monoxide diffusion capacity; VAS, Visual Analog Scale.

† Data available for 71 patients.

‡ Data available for 69 patients.

**Supplementary Table 3** Correlation between baseline anti-Jo-1 levels and disease activity in patients with ILD at baseline and without ILD at baseline

| Parameters | ILD at baseline (n=70) | | Without ILD at baseline (n=45) | |
| --- | --- | --- | --- | --- |
| Jo-1 titers | r | *p* value | r | *p* value |
| Age of onset | -0.218 | 0.070 | -0.141 | 0.355 |
| Duration | -0.001 | 0.996 | -0.118 | 0.439 |
| PGA VAS | 0.127 | 0.296 | -0.024 | 0.875 |
| Muscle VAS | 0.151 | 0.212 | -0.084 | 0.582 |
| Pulmonary VAS | 0.050 | 0.682 | 0.069 | 0.654 |
| Skin VAS | 0.247 | 0.039 | 0.090 | 0.558 |
| Joint VAS | 0.283 | 0.018 | -0.201 | 0.184 |
| ALT(0-40 IU/L) | -0.029 | 0.811 | 0.209 | 0.173 |
| AST(0-40IU/L) | 0.126 | 0.297 | 0.255 | 0.094 |
| LDH(100-250IU/L) | 0.121 | 0.316 | 0.078 | 0.623 |
| CK(26-200U/mL) | 0.191 | 0.114 | 0.207 | 0.183 |
| IgG(694-1620mg/dl) | 0.281 | 0.020 | 0.053 | 0.738 |
| IgA(68-378mg/dl) | 0.270 | 0.026 | -0.014 | 0.931 |
| IgM(60-263mg/dl) | 0.089 | 0.469 | 0.311 | 0.043 |
| ESR(0-20mm/h) | 0.254 | 0.039 | 0.094 | 0.550 |
| CRP(＜0.8mg/dl) | 0.187 | 0.129 | 0.219 | 0.154 |
| Fet | -0.112 | 0.502 | -0.001 | 0.994 |

PGA, Physician’s Global Assessment; VAS, Visual Analog Scale; ALT, alanine aminotransferase; AST, aspartate aminotransferase; LDH, lactic dehydrogenase; CK, creatine kinase; ESR, erythrocyte sedimentation rate; CRP, C reactive protein; Fet, ferritin.

**Supplementary Table 4** Correlation between baseline anti-Jo-1 levels and disease activity in patients with and without anti-Ro-52

| Parameters | **Ro-52+(n=60)** | | **Ro-52-(n=55)** | |
| --- | --- | --- | --- | --- |
| Jo-1 titers | r | *p* value | r | *p* value |
| Age of onset | -0.237 | 0.082 | -0.237 | 0.082 |
| Duration | -0.087 | 0.525 | -0.087 | 0.525 |
| PGA VAS | 0.065 | 0.639 | 0.065 | 0.639 |
| Muscle VAS | -0.016 | 0.906 | -0.016 | 0.906 |
| Pulmonary VAS | 0.169 | 0.219 | -0.028 | 0.841 |
| Skin VAS | 0.203 | 0.138 | 0.169 | 0.219 |
| Joint VAS | 0.203 | 0.138 | 0.203 | 0.138 |
| ALT(IU/L) | 0.216 | 0.116 | 0.216 | 0.116 |
| AST(IU/L) | 0.287 | 0.035 | 0.287 | 0.035 |
| LDH(IU/L) | 0.025 | 0.859 | 0.025 | 0.859 |
| CK(U/mL) | 0.239 | 0.085 | 0.239 | 0.085 |
| IgG(mg/dl) | 0.251 | 0.073 | 0.251 | 0.073 |
| IgA(mg/dl) | 0.182 | 0.196 | 0.182 | 0.196 |
| IgM(mg/dl) | 0.397 | 0.004 | 0.397 | 0.004 |
| ESR(mm/h) | 0.230 | 0.104 | 0.230 | 0.104 |
| CRP(mg/dl) | 0.173 | 0.215 | 0.173 | 0.215 |
| Fet | 0.036 | 0.858 | 0.036 | 0.858 |

PGA, Physician’s Global Assessment; VAS, Visual Analog Scale; ALT, alanine aminotransferase; AST, aspartate aminotransferase; LDH, lactic dehydrogenase; CK, creatine kinase; ESR, erythrocyte sedimentation rate; CRP, C reactive protein; Fet, ferritin.

**Supplementary Table 5** Correlation between baseline anti-Jo-1 levels and disease activity in treated and treatment naïve patients

| Parameters | **Treated(n=72)** | | **Treatment naive(n=43)** | |
| --- | --- | --- | --- | --- |
| Jo-1 titers | r | *p* value | r | *p* value |
| Age of onset | -0.323 | 0.006 | -0.263 | 0.088 |
| Duration | 0.028 | 0.813 | -0.033 | 0.836 |
| PGA VAS | 0.060 | 0.616 | 0.134 | 0.392 |
| Muscle VAS | 0.101 | 0.398 | 0.152 | 0.329 |
| Pulmonary VAS | 0.074 | 0.535 | -0.241 | 0.120 |
| Skin VAS | 0.235 | 0.047 | 0.306 | 0.046 |
| Joint VAS | 0.138 | 0.249 | 0.221 | 0.154 |
| ALT(0-40 IU/L) | 0.075 | 0.534 | 0.022 | 0.887 |
| AST(0-40IU/L) | 0.162 | 0.177 | 0.215 | 0.167 |
| LDH(100-250IU/L) | 0.048 | 0.695 | 0.130 | 0.406 |
| CK(26-200U/mL) | 0.210 | 0.081 | 0.331 | 0.030 |
| IgG(694-1620mg/dl) | 0.215 | 0.078 | 0.310 | 0.043 |
| IgA(68-378mg/dl) | 0.085 | 0.488 | 0.321 | 0.036 |
| IgM(60-263mg/dl) | 0.250 | 0.040 | 0.103 | 0.512 |
| ESR(0-20mm/h) | 0.218 | 0.073 | 0.205 | 0.204 |
| CRP(＜0.8mg/dl) | 0.212 | 0.081 | 0.183 | 0.247 |
| Fet | -0.070 | 0.713 | 0.010 | 0.959 |

PGA, Physician’s Global Assessment; VAS, Visual Analog Scale; ALT, alanine aminotransferase; AST, aspartate aminotransferase; LDH, lactic dehydrogenase; CK, creatine kinase; ESR, erythrocyte sedimentation rate; CRP, C reactive protein; Fet, ferritin.

**Supplementary Table 6** Longitudinal association between anti-Jo-1 autoantibody levels and disease activity in patients with ASS

| Parameters | Overall(n=43) | | ILD at baseline (n=22) | | No ILD at baseline (n=21) | |
| --- | --- | --- | --- | --- | --- | --- |
| Jo-1 titers | 𝛃 | *p* value | 𝛃 | *P* value | 𝛃 | *p* value |
| PGA VAS | 0.002 | 0.001 | 0.002 | 0.003 | 0.001 | 0.001 |
| Muscle VAS | 0.0003 | <0.0001 | 0.001 | 0.052 | 0.001 | <0.0001 |
| Pulmonary VAS | 0.002 | 0.013 | 0.003 | 0.006 | 0.001 | 0.001 |
| Skin VAS | <0.0001 | 0.366 | 4.827E-5 | 0.946 | <0.0001 | 0.456 |
| Joint VAS | <0.0001 | 0.574 | 0.001 | 0.015 | <0.0001 | 0.407 |
| ALT(IU/L) | 0.014 | 0.113 | -0.005 | 0.052 | 0.023 | <0.0001 |
| AST(IU/L) | 0.010 | 0.033 | 0.008 | 0.279 | 0.009 | 0.240 |
| LDH(IU/L) | 0.025 | 0.431 | 0.013 | 0.837 | 0.022 | 0.406 |
| CK(U/mL) | 1.328 | 0.016 | 0.183 | 0.312 | 1.984 | <0.0001 |
| IgG(mg/dl) | 0.482 | <0.0001 | 0.540 | 0.002 | 0.384 | <0.0001 |
| IgA(mg/dl) | 0.051 | 0.198 | 0.123 | <0.0001 | 0.039 | 0.041 |
| IgM(mg/dl) | 0.079 | 0.001 | 0.090 | 0.028 | -0.004 | 0.935 |
| ESR (mm/h) | 0.002 | 0.764 | 0.009 | 0.072 | -0.005 | 0.565 |
| CRP (mg/dl) | 0.001 | 0.052 | 0.001 | 0.095 | 0.001 | 0.189 |
| %FVC | -0.025 | <0.0001 | -0.045 | 0.004 | -0.021 | 0.001 |
| %FEV1 | -0.021 | <0.0001 | -0.035 | 0.004 | -0.019 | 0.002 |
| %DLco | -0.011 | 0.074 | -0.021 | <0.0001 | -0.010 | 0.113 |

Abbreviation: PGA, Physician’s Global Assessment; VAS, Visual Analog Scale; ALT, alanine aminotransferase; AST, aspartate aminotransferase; LDH, lactic dehydrogenase; CK, creatine kinase; ESR, erythrocyte sedimentation rate; CRP, C reactive protein; %FVC, percent predicted forced vital capacity; %FEV1, percent predicted forced expiratory volume in one second; %DLco, percent predicted carbon monoxide diffusion capacity.

**Supplementary Table 7** Area under ROC of univariate and multivariate COX model for death

| Variables | Area under the ROC curve | *p* value | 95% CI |
| --- | --- | --- | --- |
| Age at onset, yrs | 0.742 | 0.009 | 0.588-0.895 |
| Arthritis at baseline | 0.684 | 0.047 | 0.531-0.838 |
| IgA(mg/dl) | 0.630 | 0.179 | 0.392-0.869 |
| CRP (mg/dl) | 0.691 | 0.039 | 0.518-0.865 |
| Age at onset and CRP | 0.770 | 0.004 | 0.625-0.915 |

Abbreviation: ROC, receiving operating characteristics; CRP, C reactive protein,


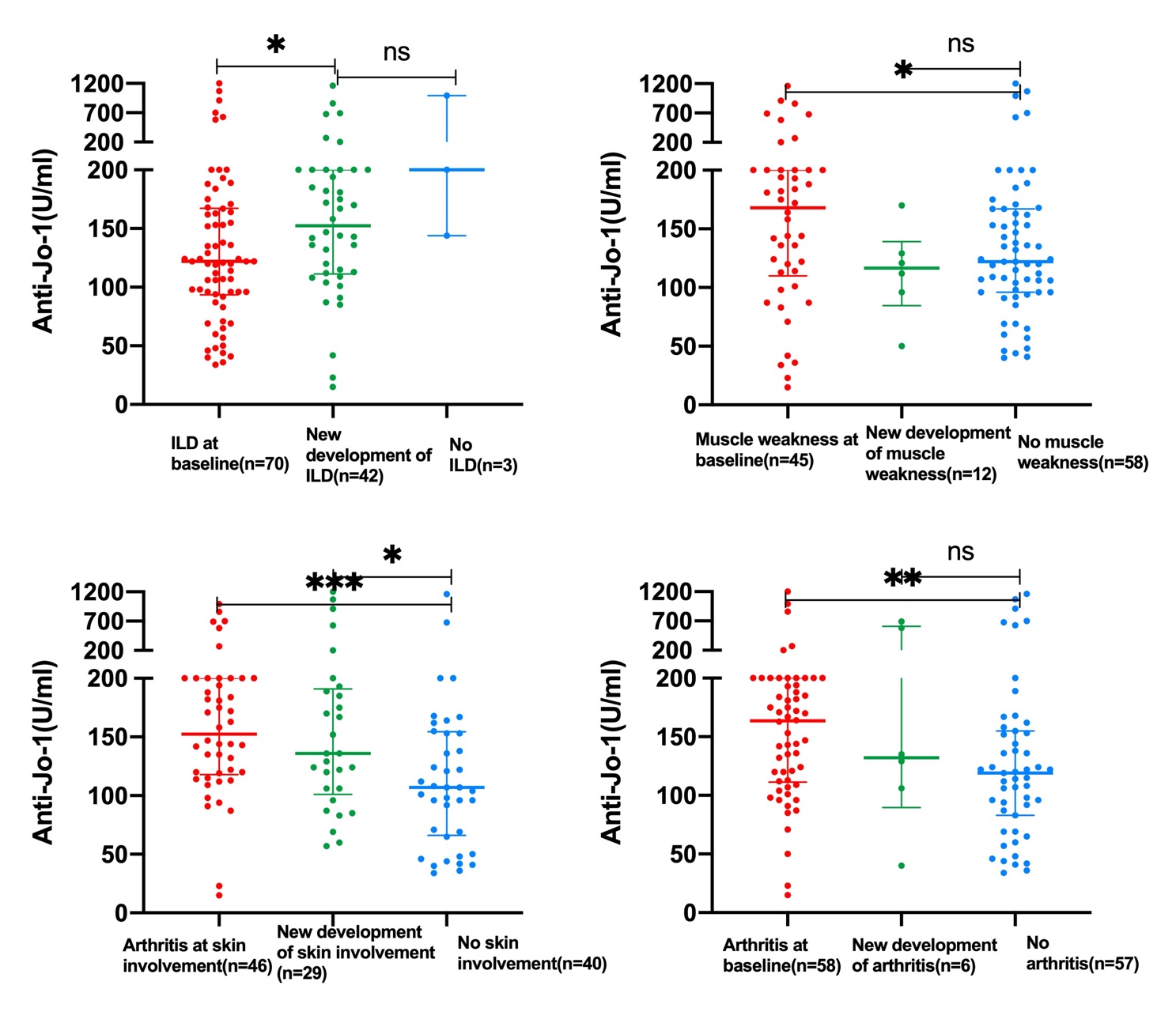


**Figure 1 The serum levels of anti-Jo-1 in patients with anti-synthetase syndrome.** (A) Anti-Jo-1 levels in patients with ILD at baseline, new development of ILD, and no ILD ; (B) Anti-Jo-1 levels in patients with muscle weakness at baseline, new development of muscle weakness, and no muscle weakness; (C) Anti-Jo-1 levels in patients with skin involvement at baseline, new development of skin involvement, and no skin involvement; (D) Anti-Jo-1 levels in patients with arthritis at baseline, new development of arthritis, and no arthritis. **p* < 0.05; ***p* < 0.01.
