# Supplementary figures and images for: Clinical and prognostic associations of anti-Jo-1 antibody levels in patients with antisynthetase syndrome
Source: Respir Res. 2024 May 29;25:222. doi: 10.1186/s12931-024-02851-w (PMC11137886; doi:10.1186/s12931-024-02851-w)

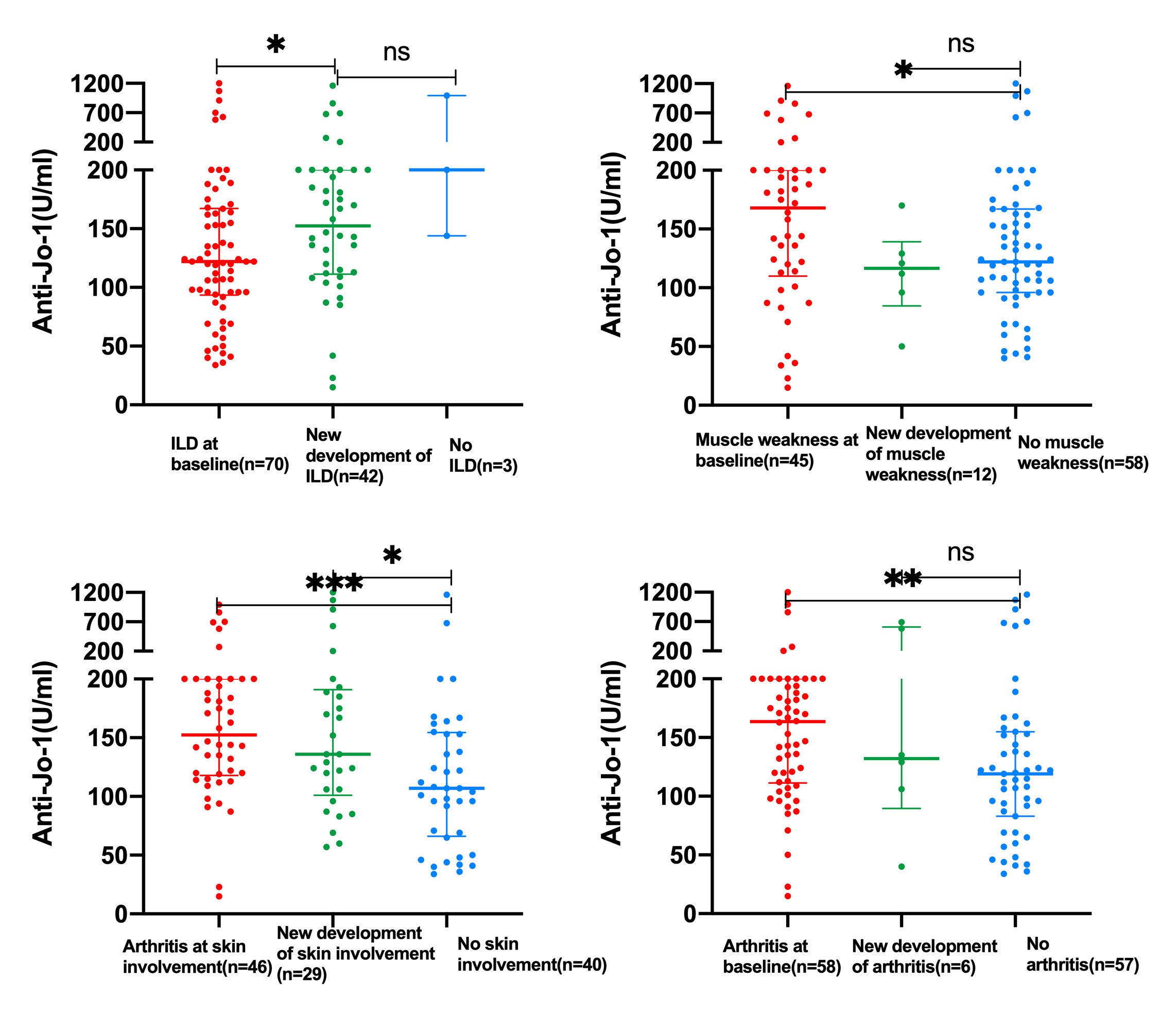

Supplement: Supplementary file 2 — Supplementary Material 2. [file 12931_2024_2851_MOESM2_ESM.jpg]
